# Plant metabolomics reveals changes in the composition of *Tripterygium wilfordii* Hook F processed by liquorice

**DOI:** 10.3389/fchem.2026.1770318

**Published:** 2026-02-26

**Authors:** Quan Rao, Xiao-Hua Fu, Cong-En Zhang, Guang-Chao Ma, Xiao-Hong Yu, Hao Wu, Zhi-Jie Ma

**Affiliations:** 1 Department of General Surgery, Beijing Friendship Hospital, Capital Medical University, Beijing, China; 2 Beijing Friendship Hospital, Capital Medical University, Beijing, China; 3 The First Affiliated Hospital, Jinan University, Guangzhou, China; 4 Department of Anesthesiology, Pain and Perioperative Medicine, The First Affiliated Hospital of Zhengzhou University, Zhengzhou, China; 5 Department of Pharmacy, Beijing Ditan Hospital, Capital Medical University, Beijing, China

**Keywords:** compositional changes, liquorice, metabolomics, processed, *tripterygium wilfordii*

## Abstract

**Background:**

*Tripterygium wilfordii* Hook F (TwHF), a traditional Chinese herb with immunosuppressive activity, has demonstrated clinical efficacy in the treatment of autoimmune diseases. However, the clinical application of TwHF has been greatly limited owing to its toxicity. Previously, we showed that the toxicity of TwHF can be reduced by liquorice processing, but the material basis for this reduced toxicity remains unclear.

**Methods:**

Here, we hypothesized that liquorice processing affects the components of TwHF. And LC-IT-TOF/MS together with plant metabolomics was applied to analyse the chemical composition of raw TwHF (Raw), TwHF combined with liquorice (Com), and TwHF processed by liquorice (Pro).

**Results:**

As a result, three differential compounds in TwHF, including triptolide, celastrol and wilforlide A, were tentatively identified. At the same time, we found that there were nine differential compounds from liquorice, including isoliquiritin, uralenin, coumestrol, liquiritigenin, schaftoside, glycyrrhizic acid, glyyunnanprosapogenin D, uralsaponin B and isolicoflavonol.

**Conclusion:**

These results will provide a basis for the scientific and rational use of TwHF processed by liquorice in the clinic.

## 1 Introduction

*Tripterygium wilfordii* Hook F (TwHF), a traditional Chinese herb, has been widely used clinically for the treatment of inflammatory and autoimmune diseases (Bao and Dai, 2011). It has demonstrated efficacy in the treatment of rheumatoid arthritis (RA), systemic lupus erythematosus (SLE), immune complex nephritis, and other diseases of the autoimmune system (Li et al., 2015; Xu et al., 2016; Zhang et al., 2016). However, the clinical application of TwHF has been greatly limited by its toxicity, including hepatotoxicity, nephrotoxicity, and reproductive toxicity (Marcus, 2014; Wan et al., 2014; Zhang et al., 2014).

There is only limited knowledge of the methods of attenuating TwHF toxicity through processing. This includes research on the methods of modern processing such as net processing, steaming, and the addition of auxiliary materials (sheep blood). Presently, TwHF undergoes net processing for clinical application, and the xylem, which has the lowest toxicity, is used medicinally; however, the toxicity and adverse reactions remain prominent (Cao et al., 2015; Cao et al., 2016). Recent studies have shown that liquorice exerts protective effects on the liver (Gong et al., 2015; Zhang et al., 2016), and its main component is glycyrrhizic acid (Cao et al., 2015; Chirumbolo, 2016). Our research group has investigated TwHF processed by liquorice (Zhao et al., 2017). In a preliminary mechanism analysis, the toxicity of TwHF was greatly reduced after processing by liquorice (Zhao et al., 2016). However, there have been no studies on changes in the chemical composition of TwHF before and after processing.

TwHF has a wide range of chemical constituents, and more than 100 ingredients have been identified to date; these include diterpenoids, triterpenoids, sesquiterpenoids, and alkaloids. Studies have shown that triptolide, a diterpenoid, is the most active epoxy diterpenoid lactolide compound in TwHF (Li et al., 2014); however, it is also the main cause of toxicity due its toxic side effects in the liver, kidney, and reproductive system (Wang et al., 2016; Xi et al., 2017). Celastrol, the triterpenoid component, was the first monomer isolated from TwHF (Greenhill, 2015; Liu et al., 2015). Although it has various pharmacological activities, celastrol is the main toxic component of TwHF. Alkaloids have clear immunosuppressive effects and are effective for the treatment of rheumatoid arthritis; however, wilfortrine and euonine are also toxic (Li et al., 2015; Marcus, 2014; Wan et al., 2014; Xu et al., 2016). We previously showed that the content of triptolide and other components in TwHF was closely related to its hepatotoxicity. Based on this, we hypothesized that the material basis of TwHF processed by liquorice may be related to changes in the toxic components after processing.

In this study, LC-IT-TOF/MS correlation analysis was carried out on TwHF obtained from different habitats, and the differential components were identified by high-resolution mass spectrometry and combined with the multivariate statistical method of metabolomics (Lv et al., 2015; Shi et al., 2016; Zhao and Hartung, 2015). To evaluate the changes in TwHF composition before and after processing, we analysed the composition of TwHF and liquorice. The influence of the processing process was analysed and revealed differences in the composition of TwHF before and after processing, providing a reference for the rational use of TwHF in the clinic.

## 2 Materials and methods

### 2.1 Chemicals and reagents

Acetonitrile and methanol (HPLC grade) were purchased from Fisher (United States and Trinidad, respectively). Formic acid was purchased from Sigma Aldrich Co. (St Louis, United States). Double-distilled water was purified using a Millipore water purification system (Millipore, Bedford, MA). Other chemicals were of analytical grade. Liquorice (*Glycyrrhiza uralensis* Fisch., Batch number SAA11) was collected from Neimenggu Province, China and from the different habitats of TwHF (Table 1). The herb was authenticated by Professor Kui-Jun Zhao (Capital Medical University, Beijing, China).

**TABLE 1:** Batches of *Tripterygium wilfordii* samples obtained from different habitats.

Sample no.	Habitat	Primitive plant	Lots
1	YunNan	*Tripterygium wilfordii*	170,903
2	HuBei	*Tripterygium wilfordii*	17,082,102
3	AnHui	*Tripterygium wilfordii*	180,104
4	FuJian	*Tripterygium wilfordii*	171,112
5	HeNan	*Tripterygium wilfordii*	17,121,001
6	SiChuan	*Tripterygium wilfordii*	170,921
7	ZheJiang	*Tripterygium wilfordii*	180,221
8	GuangXi	*Tripterygium wilfordii*	180,123

### 2.2 Sample preparation and quality control

#### 2.2.1 Method of extracting raw TwHF

First, 12 g samples of TwHF from different habitats (Table 1) were weighed and broken into fine granules by a powdering machine. Then, eight-times the volume of pure water was added to fully wet the samples that were soaked for 1 h, boiled, condensed, and refluxed for extraction over 45 min, by using three layers of gauze filter while hot. Then, six-times the volume of pure water was added to the filter residue reflux and extracted for 30 min. The filtrate from two filtrations was combined. The filtrate was condensed to a set volume by a rotary evaporator and then freeze-dried to form a powder. Samples from each habitat were processed independently in triplicate for each treatment group (Raw, Com, Pro).

#### 2.2.2 Preparation of TwHF combined with liquorice

Samples of TwHF (12 g) from different habitats were weighed, and 2 g raw liquorice was added to each sample in the same container. Then, the sample was broken into fine granules by a powdering machine. Eight-times the volume of pure water was added to fully wet the samples that were soaked for 1 h, boiled, condensed, and refluxed for extraction over 45 min, by using three layers of gauze filter while hot. Six-times the volume of pure water was added to the filter residue reflux for extraction over 30 min. The filtrate from two filtrations was combined. Then, the filtrate was condensed to a set volume by using a rotary evaporator and freeze-dried to powder.

#### 2.2.3 Preparation of TwHF processed by liquorice

Raw liquorice (16 g) was weighed, soaked in eight-times the volume of pure water for 1 h, decocted for 30 min, and filtered while hot. Then, the sample was boiled twice and the filtrate was combined and concentrated to a suitable volume (to fully soak the TwHF). The sample was then divided into eight even parts, the TwHF was soaked for 30 min until it was absorbed by the herbs, stir-fried at 100 °C for 10 min, to obtain the processed products of TwHF. Samples of TwHF processed by liquorice products were prepared following the method used to extract and prepare TwHF (2.1.1).

### 2.3 Chromatography and mass spectrometry conditions

The metabolic profiles of the biofluids were analysed via LC-IT-TOF/MS (Shimadzu Japan). The sample sequence was random and 5 μL of each sample was injected onto an Inertsil ODS-SP C18 (4.6 × 150 mm, 5 μm) column at a column temperature of 40 °C. The mobile phase of solvent A (water with 0.1% formic acid) and solvent B (acetonitrile with 0.1% formic acid) was separated by a 50 min linear gradient. The following gradient was used: a linear gradient of 3% B over an initial 3.0 min, 3%–15% B over 3.0–8.0 min, 15%–25% B over 8.0–15.0 min, 25%–40% B over 15.0–25.0 min, 40%–50% B over 25.0–40.0 min, 50%–75% B over 40.0–45.0 min, and 75%–100% B over 45.0–50.0 min. The flow rate was set as 1 mL/min. The detection wavelength was 275 nm, and the eluent was directly introduced into the mass spectrometer. To ensure the stability and repeatability of the system, 10 μL of each sample was combined to form a quality control (QC) sample, and every five samples were inserted and analysed.

For mass spectrometry, LC-IT-TOF/MS with an electrospray ionization source (ESI) in both positive and negative mode was used. The combined use of both positive and negative ionization LC-MS offers more comprehensive metabolome coverage than the use of a single polarity. The electrospray source parameters were fixed as follows: electrospray capillary voltage 3.5 kV in negative ionization mode and 4.5 kV in positive ionization mode. The mass range was set from m/z 50 to 1,200. The gas temperature was 200 °C in negative ionization mode and 225 °C in positive ionization mode. Gas flow was 1.5 L/min. Nozzle voltage was 500 V in both negative and positive mode.

### 2.4 Data processing and statistical methods

The initial and final retention times were set for data collection. Data were imported into Metabolists 4.0 (http://www.MetaboAnalyst.ca/) online software to estimate missing values and to filter and normalize data. Then, the obtained data matrix was introduced into SIMCA-P+14.0 (Umetrics, Umeå, Sweden) software for multivariate statistical analysis of principal component analysis (PCA) and orthogonal partial least-squares discriminant analysis (OPLS-DA). Prior to PCA, all variables obtained from the data matrix were centred on the mean and scaled to the variance. The PCA score plot was used to present a natural correlation between the observations. To identify differential compounds, the OPLS-DA model was used to explore differences in depth between raw TwHF (Raw), TwHF combined with liquorice (Com), and TwHF processed by liquorice (Pro). The OPLS-DA model with VIP values (VIP ≥1.0) and |*p*(corr)| ≥ 0.5 was selected as a differential compound. Data were analysed using the SPSS software program (version 22.0, Chicago, IL, United States). One-way analysis of variance (ANOVA) used a post-hoc test followed by a test to assess significant differences in the results (the difference was considered statistically significant when *p* < 0.05 and highly significant when *p* < 0.01).

## 3 Result

### 3.1 Heat map and principal component analysis

**FIGURE 1:**
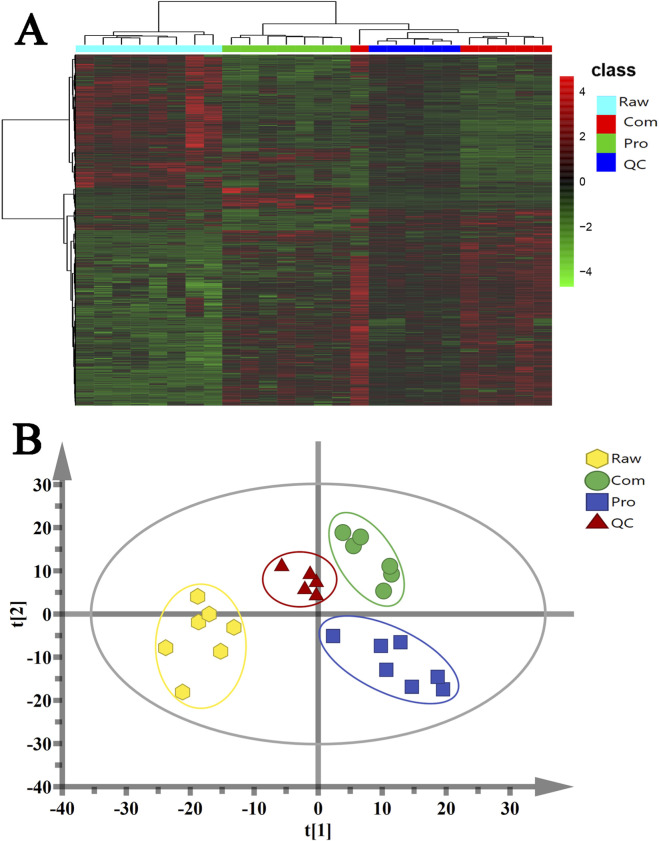
**(A)** Hierarchical clustering heatmap for the Raw, Com, Pro, and QC groups; the colour of each section is proportional to the significance of the change in metabolites (red, upregulated; green, downregulated). **(B)** Principal component analysis (PCA) score plot comparing the Raw, Com, Pro, and QC groups. QC indicates the quality control group.

Three batches of different TwHF samples were analysed, and multivariate analysis of the data matrix in the chromatogram was performed using the MetaboAnalyst (Figure 1A). shows the clustering results in the form of heat maps, with each row representing the expression level of each gene in different samples, and each column indicating the expression level of all genes in each sample. The upper tree diagram shows the results of cluster analysis for different samples from different experimental groups. The results of the cluster analysis for different genes from different samples are presented on the left tree diagram. There were significant differences in the Raw, Com, QC, and Pro groups. The expression patterns observed in the Com and Pro groups were similar, demonstrating that the sample was reproducible. PCA was used as an unsupervised statistical method to analyse metabolic differences between the Com, Pro, and Raw groups. (Figure 1B) shows a score map of the PCA derived from data obtained by the ESI mode. The QC samples were tightly clustered in the PCA score plot, demonstrating the stability of the LC-MS system throughout the analysis. In addition, in the PCA model, a clear separation trend was observed between the Com, Pro, and Raw groups, indicating considerable metabolic differences between the three groups.

### 3.2 Exploration, selection, and preliminary identification of differential compounds

**FIGURE 2:**
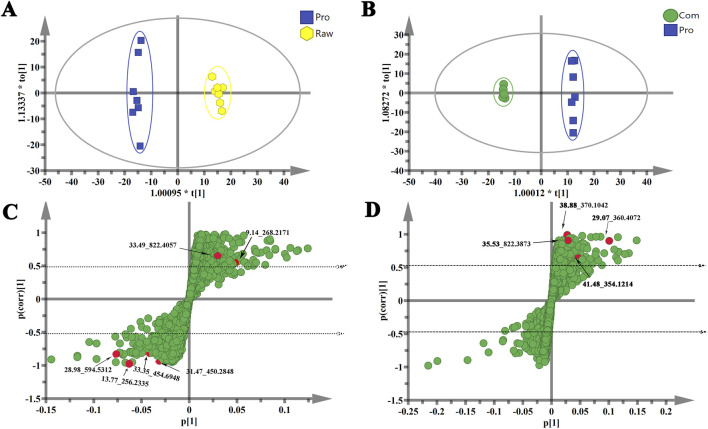
Orthogonal partial least-squares discriminant analysis (OPLS-DA) score plots showing pair-wise comparisons between the Raw and Pro **(A)** and the Pro and Com **(B)** groups. S-plot of the OPLS-DA model for the Raw and Pro **(C)** and the Pro and Com **(D)** groups. The axes on the S-plot from the predictive component are *p*1 vs. *p*(corr)1, representing the magnitude (modelled covariation) and reliability (modelled correlation) respectively. The points in red indicate the differential compounds.

To highlight the differences between groups and facilitate the subsequent search for differential compounds, the data were analysed using OPLS-DA. The projection variable weight value (VIP) reflects the contribution of metabolites to the grouping. Variables in the S-Plot far from the origin with a VIP value > 1 were considered to have a significant contribution to the grouping (Chen et al., 2016; De Preter, 2015; Wang and Wu, 2015). Differential compounds were selected based on VIP >1.0 and |p(corr)| ≥0.5 in OPLS-DA models, followed by significance testing (p < 0.05). These variables were pre-selected as differential compounds. To reduce the risk of false positives in differential compound selection, the variable |p(corr)|select ≥0.5 was the most relevant to the OPLS-DA discriminant score (Xie et al., 2018; Yan and Shen, 2015; Zhang et al., 2015). Figures 2A–D shows the results of the OPLS-DA model derived from ESI + analysis data. Figures 2A,C show the results of the OPLS-DA model using data from the Raw and Pro groups. As shown in (Figure 2A), the score map shows good model adaptability and the Raw group can be clearly separated from the Pro group. Similarly, the OPLS-DA model was constructed based on Com and Pro data (Figure 2B), and the Com group could be clearly separated from the Pro group. Figures 2C,D present the S-plots of the two models. By applying this analysis process, the variables responsible for group separation were selected as differential compound candidates.

**FIGURE 3:**
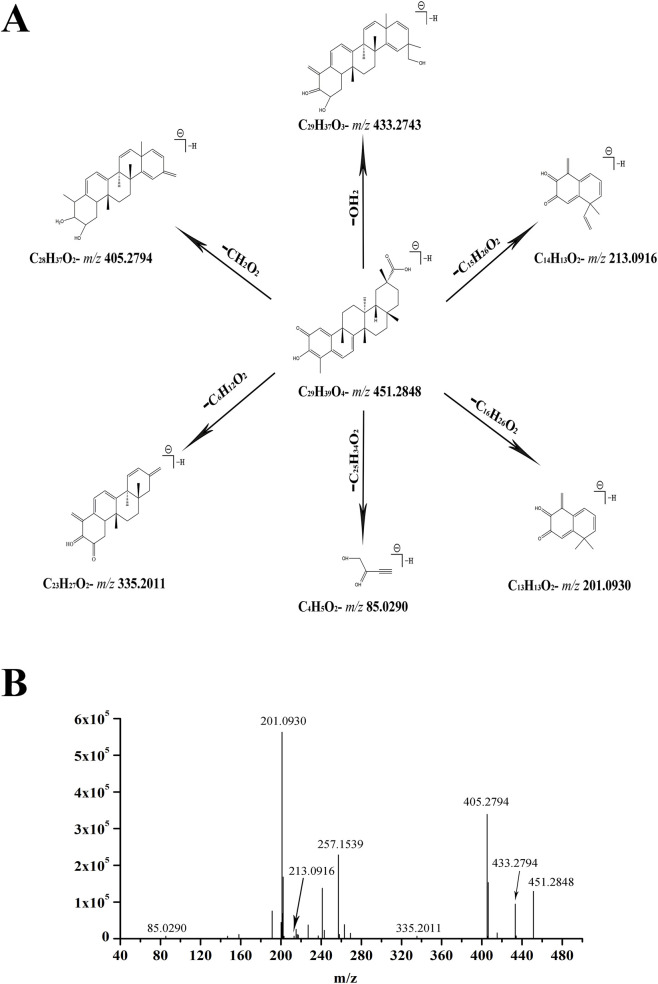
Mass spectra and mass fragment information for celastrol. The proposed MS fragmentation mechanism **(A)** and product ion spectrum of celastrol in ESI + mode **(B)**.

Next, the metabolites (*p* < 0.05) that were significantly different in the Com and Raw groups compared with the Pro group, were selected as candidate compounds. The metabolites in the ESI+ and ESI-mode analyses were combined and molecular formulas further identified. The exact mass-to-charge ratio of all compounds was initially determined by the online METLIN database. (http://www.metlin.scipps.edu/). To determine the underlying structure of the ions, targeted MS/MS analysis was applied to identify metabolites. Metabolites were identified using high-resolution MS and MS/MS fragments and database analysis. To identify metabolites, we used ions of 31.47–451.2848 with a retention time of 31.47 min and a molecular weight of 451.2848 as an example. Based upon analysis of its element composition and fractional isotope abundance, the molecular formula was speculated to be C_29_H_39_O_4_. In the positive ion spectrum, the main fragment ions screened by MS/MS analysis were observed at m/z 433.2743, 405.2794, 335.2011, 213.0916, 201.0930, and 85.0290, which may refer to lost -OH_2_, -CH_2_O_2_, -C_6_H_12_O_2_, -C_15_H_26_O_2_, -C_16_H_26_O_2_, and -C_25_H_34_O_2_. Finally, we used the online METLIN database to define its structure and to temporarily identify metabolites as celastrol. The proposed fragmentation pathway is shown in (Figure 3A) and the mass spectrum is shown in (Figure 3B). Using the above protocol, twelve differential compounds were identified, including four metabolites from the ESI + analysis and eight metabolites from the ESI- analysis, which are listed in (Tables 2, 3).

**TABLE 2:** Differentially identified metabolites for discrimination among the raw TwHF (Raw), TwHF combined with liquorice (Com), and TwHF processed by liquorice (Pro) groups.

tR (min)	Metabolite	Com vs. pro			Raw vs. pro		
		VIP	Fold	p^a^	VIP	Fold	p^a^
Data from the ESI+ mode							
31.47	Celastrol	1.16	0.08	0.006	1.39	0.12	0.008
33.25	Isoliquiritin	2.30	0.08	0.040	1.70	3.17	0.647
33.35	Wilforlide A	1.87	0.14	0.030	1.03	0.13	0.006
38.88	Uralenin	1.50	0.22	0.040	1.27	2.75	0.030
Data from the ESI− mode							
9.14	Coumestrol	<1	0.51	0.740	1.38	0.18	<0.001
13.77	Liquiritigenin	<1	0.72	0.690	1.42	8.76	0.020
28.98	Schaftoside	<1	2.70	0.460	1.70	0.06	0.002
29.07	Triptolide	1.06	0.36	0.250	1.42	12.38	0.029
32.17	Glyyunnanprosapogenin D	4.80	0.02	0.003	4.55	17.11	0.001
33.49	Glycyrrhizic acid	6.94	0.11	<0.001	5.20	11.01	0.033
35.53	Uralsaponin B	1.94	0.04	0.038	1.79	8.22	0.020
41.48	Isolicoflavonol	1.80	0.03	0.016	1.42	4.77	0.019

**TABLE 3:** Identification of differential compounds based on HPLC-MS/MS.

tR (min)	Identified compound	Mass (neutral)	Error (ppm)	Formulate	MS/MS fragment ions (m/z)
Data from the ESI + mode					
31.47	Celastrol	450.2848	12.32	C_29_H_38_O_4_	451.2641 [M + H]+433.2752 [M + H-OH_2_]^+^ 405.2794 [M + H-CH_2_O_2_]^+^ 335.2042[M + H-C_6_H_12_O_2_]^+^ 213.0928 [M + H-C_15_H_26_O_2_]^+^ 85.0284 [M + H-C_25_H_34_O_2_]^+^
33.25	Isoliquiritin	418.1272	10.62	C_21_H_22_O_9_	419.1353 [M + H]+401.1243 [M + H-OH_2_]+257.0832[M + H-C_6_H_9_O_5_]^+^ 137.0242[M + H-C_14_H_17_O_6_]^+^
33.35	Wilforlide A	454.6948	8.42	C_30_H_46_O_3_	455.5892[M + H]+437.6738[M + H-OH_2_]+352.5371[M + H-C_5_H_10_O_2_]^+^
38.88	Uralenin	370.1042	−5.36	C_20_H_18_O_7_	371.1242[M + H]+353.1056[M + H-OH_2_]+313.0843[M + H-C_3_H_5_O]^+^ 121.034[M + H-C_13_H_13_O_5_]^+^
Data from the ESI- mode					
9.14	Coumestrol	268.0382	8.76	C_15_H_8_O_5_	267.0282 [M-H]^–^ 241.0243 [M-H-C_2_H_3_]^–^ 223.0412 [M-H-CHO_2_]^–^
13.77	Liquiritigenin	256.0832	−11.22	C_15_H_12_O_4_	255.0643 [M-H]^–^ 237.0672 [M-H-OH_2_]^–^ 135.0432 [M-H-C_7_H_5_O_2_]^–^ 93.0472 [M-H-C_9_H_7_O_2_]^–^
28.98	Schaftoside	594.5312	−8.42	C_27_H_30_O_15_	593.4136 [M-H]^–^ 575.3049 [M-H-OH_2_]^–^
29.07	Triptolide	360.4072	7.62	C_20_H_24_O_6_	359.3873 [M-H]^–^ 341.3862 [M-H-OH_2_]^–^ 239.6813 [M-H-C_5_H_13_O_3_]^–^ 166.7842 [M-H-C_12_H_18_O_5_]^–^
32.17	Glyyunnanprosapogenin D	822.3892	14.73	C_42_H_62_O_16_	821.4085 [M-H]^–^
33.49	Glycyrrhizic acid	822.4057	10.62	C_42_H_62_O_16_	821.3873 [M-H]^–^ 803.3774 [M-H-OH_2_]^–^ 643.2796 [M-H-C_5_H_7_O_7_]^–^ 451.3301 [M-H-C_12_H_19_O_13_]^–^ 191.0172 [M-H-C_36_H_55_O_9_]^–^ 103.0403 [M-H-C_38_H_55_O_13_]^–^
35.53	Uralsaponin B	822.3873	8.86	C_42_H_62_O_16_	821.3957 [M-H]^–^ 803.3884 [M-H-OH_2_]^–^ 645.4271 [M-H-C_6_H_9_O_6_]^–^ 469.2814 [M-H-C_12_H_17_O_12_]^–^ 193.0372 [M-H-C_36_H_53_O_9_]^–^
41.48	Isolicoflavonol	354.1214	−17.68	C_20_H_18_O_6_	353.0942 [M-H]^–^ 335.1011 [M-H-OH_2_]^–^ 201.0876 [M-H-C_7_H_5_O_4_]^–^ 151.0103 [M-H-C_13_H_15_O_2_]^–^

Following the accurate mass determination and comparison with control retention behaviour and mass spectrometry data, only variables presenting significant changes were selected as differential compounds and their molecular formulas were identified. Mass spectrometry revealed 12 different variables between the Raw and Pro groups and eight between the Com and Pro groups (Table 2). All compounds were initially identified by the on-line TCM Database with an accurate mass charge (20 ppm was set to an acceptable mass error). For each mass ion, several candidates were provided by the above database. The candidate was subjected to further MS/MS experiments and the target molecule was verified by the characteristics of its fragment information. The identity was then determined based on the online database and literature.

**FIGURE 4:**
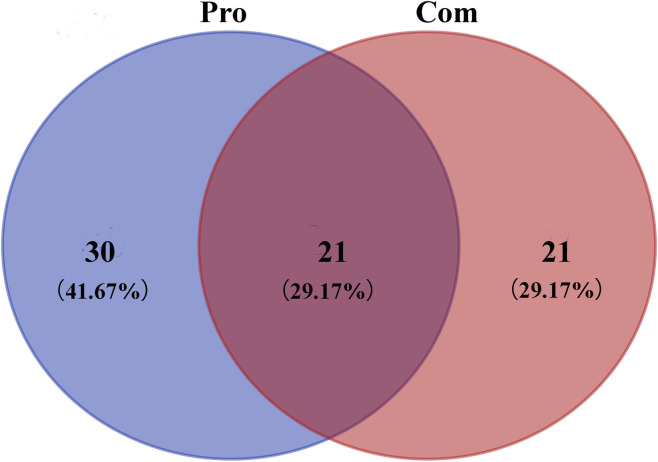
Venn diagram of compounds in *Tripterygium wilfordii* Hook F (TwHF) processed by liquorice and TwHF combined with liquorice.

The results suggested that changes in the composition of TwHF may be due to the processing method. Therefore, differential compounds following the addition of liquorice were analysed. Some compounds differed, and some were the same between the Pro and Com groups. We conducted a comprehensive analysis and identified 51 compounds in the Pro group and 42 compounds in the Com group; 21 were overlapping between the two groups (Figure 4). This indicates that there has a common role in the Pro and Com groups. In addition, some compounds differ between the two groups. This may explain the difference between the efficacy and reduced toxicity of the Pro and Com groups.

### 3.3 Changes in differential compounds of TwHF following processing by liquorice

**FIGURE 5:**
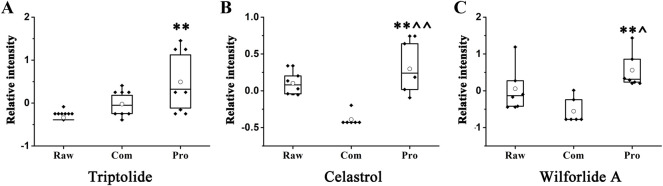
Variations in trends for metabolites that are compounds of *Tripterygium wilfordii.* **(A)** Triptolide. **(B)** Celastrol. **(C)** Wilforlide A. *, ** and *** represent *p* < 0.05, 0.01, and 0.001 compared with the Raw group, respectively. ^, ^^^, and ^^^^^ represent *p* < 0.05, 0.01, and 0.001 compared with the Com group, respectively.

In the search for changes in differential compounds caused by liquorice in the production of TwHF, we found significant (*p* < 0.05) changes in the three major chemical constituents in TwHF; triptolide, celastrol, and wilforlide A. Figure 5A shows that the triptolide content increased significantly after processing; however, there was no significant difference between the Com and Pro groups. Figure 5B shows that the content of celastrol increased significantly after processing, and there was also a significant difference between the Com and Pro groups. Figure 5C shows that the content of wilforlide A increased significantly after processing. There was also a significant difference between the Com and Pro groups. As shown in the figure, the content of celastrol and wilforlide A decreased and that of triptolide increased after combining with liquorice; however, the content of the three main components was significantly increased after being processed by liquorice.

### 3.4 Changes in differential compounds of liquorice before and after processing by liquorice

**FIGURE 6:**
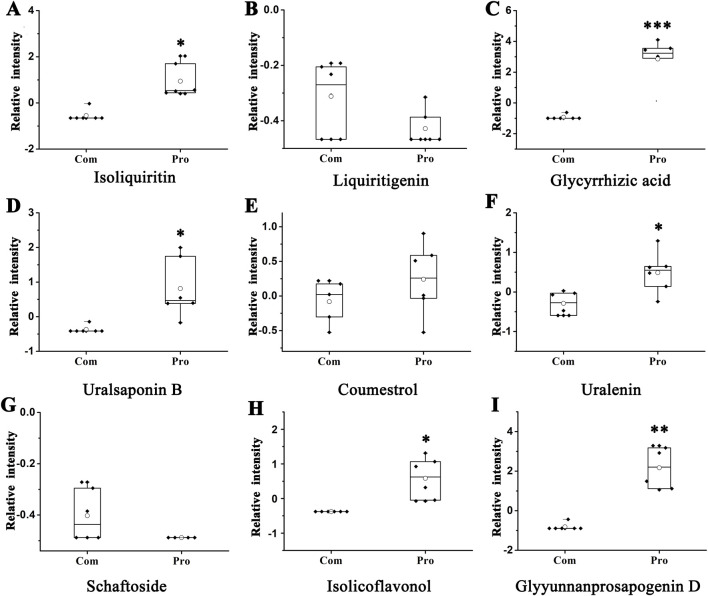
Change in the main chemical constituents of *Glycyrrhiza uralensis* before and after liquorice-processing of *Tripterygium wilfordii*. **(A)** Isoliquiritin. **(B)** Liquiritigenin. **(C)** Glycyrrhizic acid. **(D)** Uralsaponin **(B) (E)** Coumestrol. **(F)** Uralenin. **(G)** Schaftoside. **(H)** Isolicoflavonol. **(I)** Glyyunnanprosapogenin **(D)** *, **, and *** represent *p* < 0.05, 0.01, and 0.001 compared with the Com group, respectively.

Before and after processing TwHF, we found that there were significant (*p* < 0.05) changes in nine major chemical constituents in liquorice. Then, we analysed the effect of liquorice on TwHF and found (Figure 6) that isoliquiritin, glycyrrhizic acid, uralsaponin B, uralenin, isolicoflavonol, and glyyunnanprosapogenin D were significantly increased after being processed by liquorice. We analysed the main compositional changes induced by the processing process.

## 4 Discussion

TwHF was first collected in ‘Shen Nong’s Herbal Classic’. Traditionally, it has been used externally and not internally. TwHF has autoimmune effects when used within a certain dose range during clinical application. In refractory diseases, it has proven to be the most effective (Landewe and van der Heijde, 2014; Lv et al., 2014). There are no similar Chinese medicines that can replace TwHF; however, its use is accompanied by multiple organ damage, which greatly limits its clinical application (Ma et al., 2017; Xie et al., 2018). With the promotion of TwHF for clinical use, studies of liver toxicity have also increased in recent years. Since the effective dose of TwHF is close to the toxic dose and adverse effects can occur within the normal dose range, it is difficult to adjust the clinical dosage so that efficacy is exerted and toxicity is avoided.

Traditional processing technology is a significant part of traditional Chinese medicine (TCM). Employing the correct processing technology is necessary in manufacturing clinical decoction pieces. As a commonly used TCM for medicinal properties, liquorice can be used for detoxification. It is often used in combination with TwHF. Clinical practice and basic research have confirmed that liquorice can be used to increase the efficacy and reduce its toxicity. However, current research has focused on the attenuation effect of processing, and no studies have yet shown changes in the chemical composition of TwHF before and after processing.

We found that liquorice affects the main components of TwHF. Triptolide is the most active epoxy diterpene lactone compound in TwHF. It is also the main component responsible for the toxic side effects of TwHF and exerts toxicity in many systems in the body (Zhao et al., 2015; Zhou et al., 2018). The triterpenoid component celastrol, although has various pharmacological activities, is the main toxic component. Thus, the various components contained in TwHF are both medicinal and toxic; the toxicity and efficacy of TwHF are closely related and cannot be viewed on their own. Both efficacy and toxicity are based on the presence of certain substances, and toxicity should be explored based on the substances present. Therefore, we speculate that the material basis of TwHF processed by liquorice may be related to changes in toxic components after processing. Experiments have shown that toxic components, such as triptolide and celastrol, have indeed changed significantly after liquorice processing. Presently, most studies have investigated toxicity, and there is little concern about its efficacy. However, whether these changes in toxic components will affect its efficacy remains to be further studied; we aim to assess this in future research. Although triptolide content increased after processing, liquorice-derived components such as glycyrrhizic acid and isoliquiritin may exert hepatoprotective effects through Nrf2 pathway activation, potentially counterbalancing the toxicity of elevated triptolide. Further pharmacological and toxicological studies are warranted to elucidate the net effect of these compositional changes.

Triptolide was significantly upregulated in the Pro group (12.38 fold), while showing a slight increase (1.23 fold) in the Com group. Conjugated licorice-derived compounds (e.g., glycyrrhizic acid, isoliquiritagenin) were significantly upregulated (4.77–11.01 fold) in the Pro group, suggesting a potential balancing mechanism where “toxic components are upregulated while detoxifying components are simultaneously enhanced.” Moreover, licorice-derived differential compounds (e.g., glycyrrhizic acid, glycyrrhizin) exhibited significantly higher upregulation multiples in the Pro group compared to the Com group, indicating that the processing technique enhances the dissolution or transformation of licorice components. Both licorice processing and combination therapy achieve detoxification effects through alterations in chemical constituents, though their mechanisms and potency differ: The core bioactive components of Tripterygium wilfordii (e.g., triptolide, alkaloids) are crucial for its treatment of autoimmune diseases. Processing Group (Pro): In the processing group, triptolide was significantly upregulated, while components in licorice with established hepatoprotective effects were also significantly increased, achieving a dual effect of “reducing toxicity and enhancing efficacy.” Combination group (Com): Direct mixing of licorice and TwHF resulted in only slight downregulation of toxic components and limited upregulation of detoxifying components. Overall detoxification strength is weaker than the processed group. The Pro group, through targeted regulation of chemical constituents, widened the “effective dose - toxic dose” margin, facilitating clinical dose adjustment—particularly suitable for patients requiring long-term medication.

Similarly, there were significant changes in various components of liquorice after processing. This includes the main component of liquorice, glycyrrhizic acid, which has protective effects on the liver. This was mainly owing to compositional changes that occurred during processing. It is unclear whether changes in these components have any effect on the efficacy and pharmacodynamic characteristics of TwHF. Therefore, we aim to conduct further in-depth research to provide a reference for the rational use of TwHF in the clinic, which will help to improve the quality and safety evaluation of TwHF processed by liquorice. The processed group (Pro) offers significant detoxification effects and stable component composition, minimizing long-term medication safety risks. Suitable for large-scale production in major medical institutions or Chinese herbal medicine enterprises, standardized processing ensures consistent product quality, providing safe and controllable herbal slices for clinical use. The compounding group (Com) is applicable where specialized processing equipment is unavailable. Its simple, highly operable process enables rapid preliminary detoxification of TwHF, serving as a complementary approach to the processing group and offering diverse clinical options. This study provides more specific references for rational clinical application.

## Data Availability

The original contributions presented in the study are included in the article/Supplementary Material, further inquiries can be directed to the corresponding authors.
