# Correction: Plant metabolomics reveals changes in the composition of *Tripterygium wilfordii* Hook F processed by liquorice

**DOI:** 10.3389/fchem.2026.1822564

**Published:** 2026-03-31

**Authors:** Quan Rao, Xiao-Hua Fu, Cong-En Zhang, Guang-Chao Ma, Xiao-Hong Yu, Hao Wu, Zhi-Jie Ma

**Affiliations:** 1 Department of General Surgery, Beijing Friendship Hospital, Capital Medical University, Beijing, China; 2 Beijing Friendship Hospital, Capital Medical University, Beijing, China; 3 The First Affiliated Hospital, Jinan University, Guangzhou, China; 4 Department of Anesthesiology, Pain and Perioperative Medicine, The First Affiliated Hospital of Zhengzhou University, Zhengzhou, China; 5 Department of Pharmacy, Beijing Ditan Hospital, Capital Medical University, Beijing, China

**Keywords:** compositional changes, liquorice, metabolomics, processed, tripterygium wilfordii

**Affiliation** “The First Affiliated Hospital, Jinan University, Guangzhou, China” was omitted for author Xiao-Hua Fu. This **Affiliation** has now been added.

**Authors** Zhi-Jie Ma and Xiao-hua Fu were erroneously assigned to **Affiliation** “Beijing Friendship Hospital, Capital Medical University, Beijing, China”. This **Affiliation** has now been removed for **Authors** Zhi-Jie Ma and Xiao-hua Fu.

The original article has been updated.

