# Supplementary material for: Plant metabolomics reveals changes in the composition of Tripterygium wilfordii Hook F processed by liquorice
Source: Front Chem. 2026 Feb 26;14:1770318. doi: 10.3389/fchem.2026.1770318 (PMC12979392; doi:10.3389/fchem.2026.1770318)
Supplement: Supplementary file 1 [file DataSheet1.docx]

***Supplementary materials for*：**

**Plant metabolomics reveals changes in the composition of *Tripterygium wilfordii* Hook F processed by liquorice**

Quan Rao^1,2^, Cong-en Zhang^2^, Guang-chao Ma^2^, Xiao-hong Yu^2^, Zhi-jie Ma^2,3*^, Hao Wu^2*^

*^1^Department of General Surgery, Beijing Friendship Hospital, Capital Medical University, Beijing, PR China.*

*^2^ Beijing Friendship Hospital, Capital Medical University, Beijing, PR China.*

*^3^Department of Pharmacy, Beijing Ditan Hospital, Capital Medical University, Beijing, PR China.*

Correspondence:

Hao Wu,

Beijing Friendship Hospital, Capital Medical University, 95 Yong-an Road, Xi-Cheng District, Beijing 100050, China.

E-mail: wuhao906991@163.com; Fax: +86 63138748

Zhi-jie Ma,

Beijing Friendship Hospital, Capital Medical University, 95 Yong-an Road, Xi-Cheng District, Beijing 100050, China.

E-mail: [13811647091@163.com](mailto:13811647091@163.com); Tel/Fax: +86 63138562

* To whom correspondence should be addressed.

# The HPLC finger print analysis of TwHF

5 μL of each sample was injected onto an Inertsil ODS-SP C_18_ (4.6 × 150 mm, 5 μm) column at a column temperature of 40°C. The mobile phase of solvent A (water with 0.1% formic acid) and solvent B (acetonitrile with 0.1% formic acid) was separated by a 50 min linear gradient. The following gradient was used: a linear gradient of 3% B over an initial 3.0 min, 3–15% B over 3.0–8.0 min, 15–25% B over 8.0–15.0 min, 25–40% B over 15.0–25.0 min, 40–50% B over 25.0–40.0 min, 50–75% B over 40.0–45.0 min, and 75–100% B over 45.0–50.0 min. The flow rate was set as 1 mL/min. The detection wavelength was 275 nm.

Professional software “Similarity Evaluation System for Chromatographic Fingerprint of Traditional Chinese Medicine” (Version 2004A, SES software) was used for evaluating the similarities between different samples. The reference chromatogram was generated with average data.

To obtain a stable and repeatable TwHF for the quality control, a method validation of the HPLC fingerprint analyses was conducted based on the retention time and peak area. The method precision was assessed by six replicate measurements of a random selected sample. The relative standard deviations (RSD) values of retention times and peak areas of all peaks were <3 %, respectively. The repeatability of the method was assessed by analyzing six different sample solutions independently prepared from the same sample. The stability was evaluated by analyzing the same sample at 0, 2, 4, 6, 8, and 24 h after solution preparation. The RSD of the peak areas was <3 %. This indicated that the sample solution of TwHF was stable within 24 h. All results indicated that the method of HPLC fingerprint analysis was reliable and satisfactory. The HPLC fingerprints of TwHF were obtained under optimized conditions.

The fingerprint of raw TwHF was then established (**Figure S1**). **Table S1** shows the values of similarity of each sample. The closer the similarity values to 1, the more similar the chromatogram to the reference chromatogram. As is shown in **Table S1**, the similarity values of all the 8 samples was higher than 0.95, the stability of sample was proved.


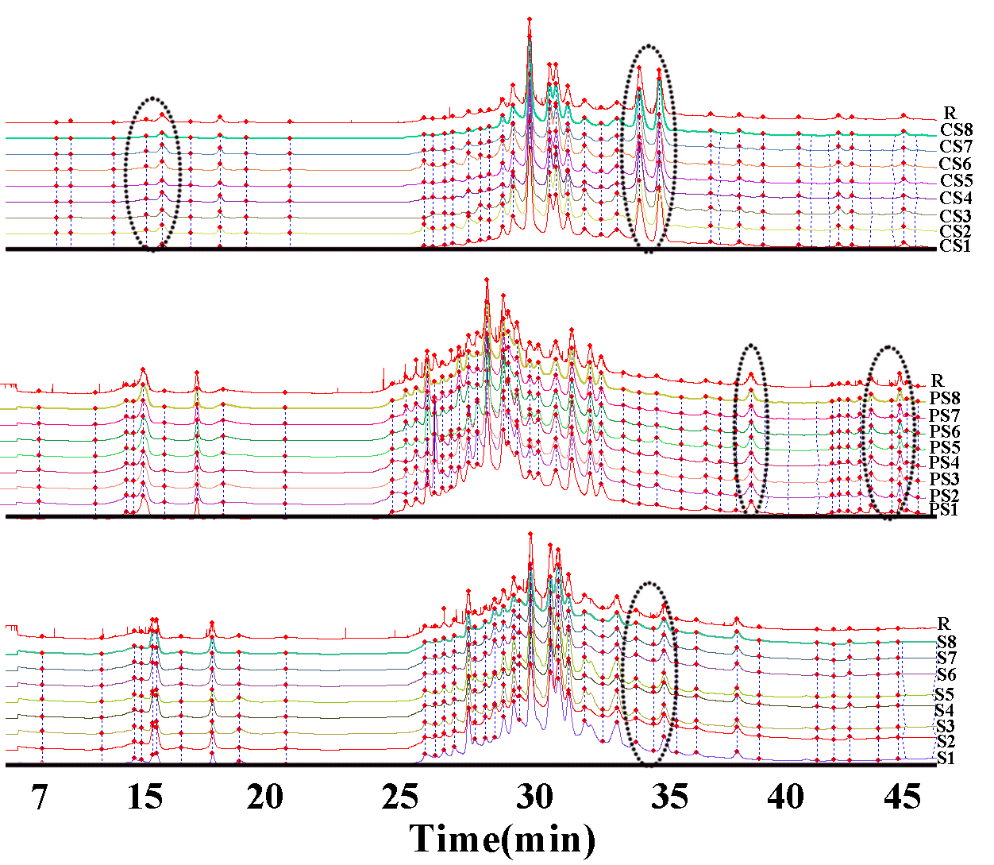


**Figure S1** HPLC fingerprints of samples of TwHF. Characteristic changes in composition of the abnormal samples were marked with black dotted circles. R: reference standard chromatographic fingerprint; S: raw TwHF; PS: TwHF processed by liquorice; CS: TwHF combined with liquorice. Ten peaks were identified and labeled with consecutive numbers. *1* wilforidine; *2* wilfornine; *3* triptophenolide; *4* wilfordine; *5* wilforgine; *6* wilforine; *7* triptolide; *8* celastrol; *9* wilforlide A; *10* wilfortrine.

**Table S1:** The similarities of chromatograms of raw TwHF.

| No. | Similarity | No. | Similarity |
| --- | --- | --- | --- |
| S1 | 0.96 | S5 | 0.98 |
| S2 | 0.98 | S6 | 0.96 |
| S3 | 0.97 | S7 | 0.97 |
| S4 | 0.97 | S8 | 0.98 |

# Total ion chromatography map of the TwHF in different groups

Three different ion graphs were obtained in the positive ion mode. **Figure S2A** presents the raw TwHF (Raw), **Figure S2B** presents the TwHF processed by liquorice (Pro), and **Figure S2C** presents the TwHF combined with liquorice (Com). The peak intensity and degree of peak aggregation in A are clearly different from those in B and C between 10 and 15 min, and the overall peak intensity of A was significantly lower in A compared with B and C. Both B and C were confirmed to be the added liquorice. The composition influenced the ion graph; at 25–30 min, the peak intensity of C was higher than that of A and B. After 40 min, the peak intensity and peak aggregation of C was clearly different to that of A and B, which may be due to the differential changes in composition before and after processing.


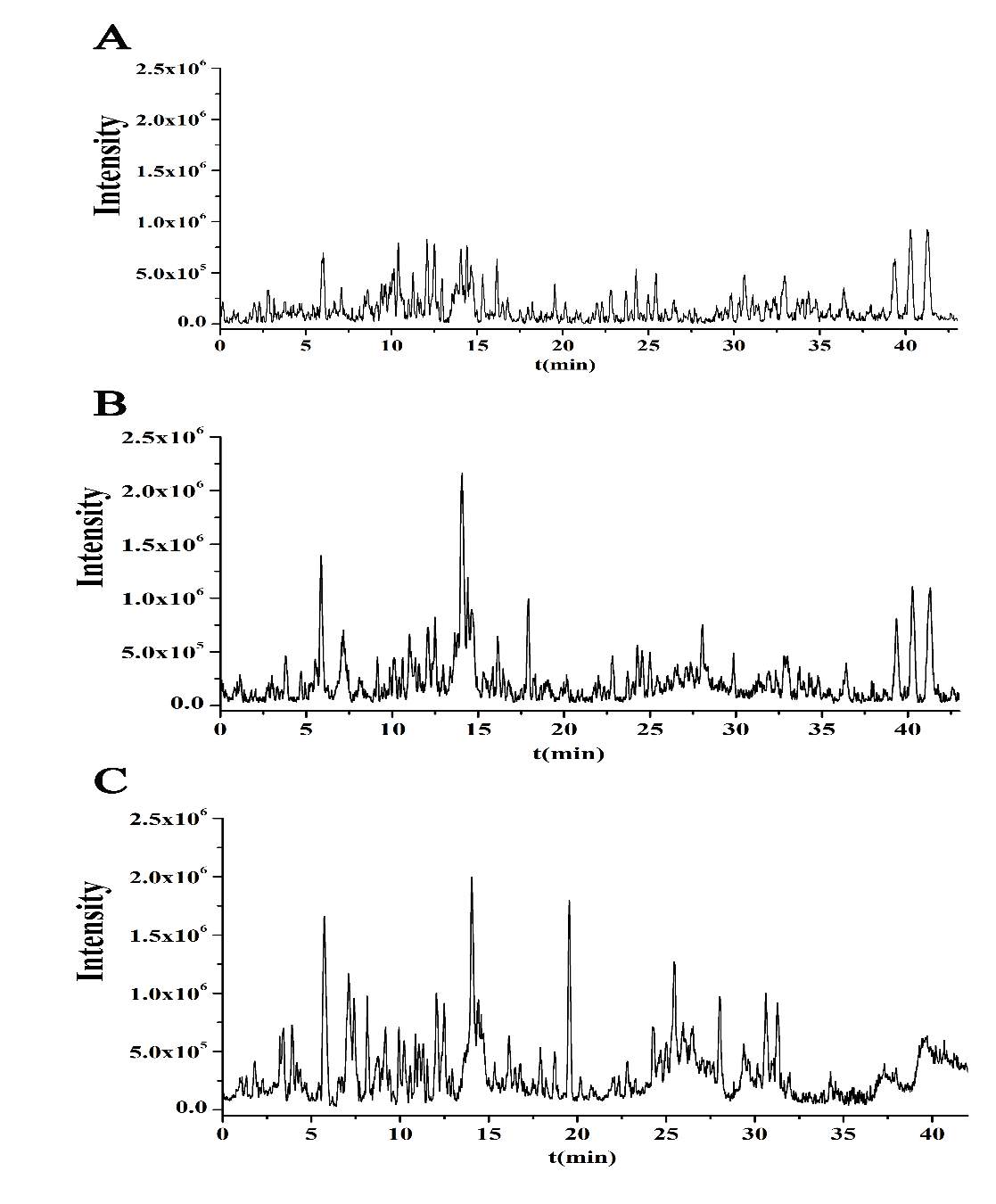


**Figure S2** Representative chromatograms of samples from the raw TwHF (Raw), TwHF combined with liquorice (Com), and TwHF processed by liquorice (Pro) groups analysed by LC-IT-TOF/MS. (A) Raw group. (B) Pro group. (C) Com group.
